# Comparative effectiveness of cortical bone trajectory screws and pedicle screws in the treatment of adjacent segment degeneration after lumbar fusion surgery: a systematic review and meta-analysis

**DOI:** 10.1186/s13018-024-04865-y

**Published:** 2024-06-28

**Authors:** Qisong Shang, Haopeng Luan, Cong Peng, Xinghua Song

**Affiliations:** Department of Spine Surgery, The Sixth Affiliated Hospital of Xinjiang Medical University, Urumqi, Xinjiang 830002 China

**Keywords:** Adjacent segment degeneration, Cortical bone trajectory, Pedicle screw, Meta-analysis, Lumbar fusion

## Abstract

**Purpose:**

To compare the efficacy and safety of cortical bone trajectory (CBT) screw and pedicle screw (PS) internal fixation in the treatment of adjacent segment degeneration (ASD) after lumbar fusion.

**Methods:**

This study was registered on International Prospective Register of Systematic Reviews (PROSPERO) (ID: CRD42023484937). We searched PubMed, Embase, Web of Science, Cochrane Library, China National Knowledge Infrastructure (CNKI), Wan Fang Database, and Wei Pu Database by computer to collect controlled clinical studies on the efficacy and safety of cortical bone trajectory (CBT) screw and pedicle screw (PS) internal fixation in the treatment of adjacent segment degeneration (ASD) after lumbar fusion from database establishment to November 2023. Two researchers screened the literature, extracted data and evaluated the risk of bias of the included studies, recorded the authors, and sample size, and extracted the intraoperative blood loss, operation time, Oswestry disability index (ODI), Visual analogue scale (VAS), disc height (DH), hospital length stay and complications in each study. Meta-analysis was performed using Revman 5.4 software provided by Cochrane Library.

**Results:**

A total of 6 cohort studies (CS) and 1 randomized controlled study with a total of 420 patients were included in this study, including 188 patients in the CBT group and 232 patients in the PS group. The CBT group had lower intraoperative blood loss than the PS group [mean difference (MD) = -129.38, 95% CI (-177.22, -81.55), *P* < 0.00001] and operation time was shorter than that of the PS group [MD = -1.42, 95% CI (-2.63, -0.20), *P* = 0.02]. Early postoperative back and leg pain improved more significantly in the CBT group [MD = -0.77, 95% CI (-1.35, -0.19), *P* = 0.01; MD = -0.24, 95% CI (-0.37, -0.10), *P* = 0.0005].

**Conclusion:**

Compared with PS, CBT for adjacent segment degeneration after lumbar fusion has the advantages of less intraoperative blood loss, shorter operation time, and less back and leg pain in the early postoperative period.

**Supplementary Information:**

The online version contains supplementary material available at 10.1186/s13018-024-04865-y.

## Introduction

Traditional pedicle screw (PS) internal fixation combined with interbody fusion is the first-line treatment for lumbar degenerative diseases, but it limits the normal movement of the spine and alters the biomechanics of adjacent segments of the fused segment, resulting in adjacent segment degeneration (ASD) [1–3]. In recent years, ASD has become a major complication after fusion, and patients inevitably experience recurrence of lower back pain and radiculopathy; at present, traditional PS internal fixation techniques combined with interbody fusion are often used to treat ASD, prolong the surgical level, perform re-decompression bone grafting and internal fixation of adjacent degenerated segments and remove the original internal fixation [4].

In 2009, Santoni et al. [5]. proposed the cortical bone trajectory (CBT) screw internal fixation technique, in which the unique external “eight” screw placement trajectory allows the screw to simultaneously pass through the three-layer cortical bone structure of the medial pedicle wall, the lateral pedicle wall, and the lateral superior vertebral wall, which has more reliable mechanical stability, less paravertebral muscle dissection, and a low probability of invasion of the facet ligament. In recent years, CBT internal fixation technology has been increasingly widely used in the treatment of lumbar degenerative diseases, with strong screw purchase, relatively less trauma, and clinical efficacy, which is also worth affirming and gradually recognized by spinal surgeons, but there are few reports on CBT internal fixation technology in the treatment of adjacent vertebral diseases. There is no previous meta-analysis related to the two. The aim of this study was to analyze and compare the clinical efficacy of CBT screw instrumentation technique with conventional PS instrumentation technique in the treatment of ASD after lumbar fusion.

## Methods

This meta-analysis followed the Cochrane handbook for conducting and the Preferred Reporting Items for Systematic Reviews and Meta-Analysis (PRISMA) guidelines for reporting [6, 7]. Two authors separately conducted literature retrieval, study eligibility, data extraction, and quality assessment with inconsistency solved by discussion and decided by the corresponding author.

### Literature search

We searched PubMed, Embase, Web of Science, Cochrane Library, China National Knowledge Infrastructure (CNKI), Wan Fang Database, and Wei Pu Database by computer to collect controlled clinical studies on the efficacy and safety of cortical bone trajectory (CBT) screw and pedicle screw (PS) internal fixation in the treatment of adjacent segment degeneration (ASD) after lumbar fusion from database establishment to November 2023. We restricted the language to English and Chinese. By preserving the literature that offered the most comprehensive information for overlapping patients, information duplication was avoided. The brief retrieval formula was “((cortical bone trajectory) OR (pedicle screw)) AND (adjacent segment degeneration)”.

### Inclusion and exclusion criteria

The inclusion criteria were as follows: (1) patients treated with CBT or PS for adjacent segment degeneration and (2) the literature reported one of the following: intraoperative blood loss, operation time, Oswestry disability index (ODI), Visual analogue scale (VAS), disc height (DH), hospital length stay, and complications; (3) All had good clinical results at initial surgery.

Exclusion criteria were as follows: (1) combined with lumbar infectious diseases, neoplastic diseases; (2) Primary surgery not fused; (3) Internal fixation breakage, infection at initial surgery; (4) review, meeting, expert opinion, case report, literature that could not obtain the full text; (5) animal experiments, in vitro/biomechanical studies.

### Literature screening and data extraction

Two researchers independently conducted a literature review, adhering to specified inclusion and exclusion criteria, and performed data extraction and cross-verification. In instances of disagreement, a resolution was sought through discussion. When necessary, a third investigator’s input was obtained, and data extraction was conducted using a structured template. The primary data elements extracted encompassed: (1) General details of the included studies, such as title, authorship, and year of publication; (2) Study demographics, including geographical location, sample size, age demographics, duration of operation, and follow-up duration; (3) Clinical outcomes of interest, covering intraoperative blood loss, surgical duration, Oswestry Disability Index (ODI), Visual Analogue Scale (VAS) for pain, Disc Height (DH), duration of hospitalization, and any postoperative complications; (4) Critical aspects of bias risk assessment, including the methodology of study population selection, group comparability, and the approaches used for the measurement of exposure variables.

### Literature quality evaluation

The bias risk assessment of the included literature was independently conducted by two evaluators and subsequently cross-verified. In cases of disagreement regarding the assessment outcomes, a third evaluator intervened to facilitate discussion and decision-making. The risk of bias was evaluated using the Cochrane Handbook’s recommended 5.4 Bias Risk Assessment Tool, which examines aspects such as sequence generation, allocation concealment, blinding, data integrity, selective reporting, and other potential biases. The risk level was categorized as either “low risk,” “high risk,” or “unclear risk.” Additionally, the Newcastle-Ottawa Scale (NOS) criteria were employed to assess the quality of cohort study (CS) literature, with articles scoring ≥ 7 considered to be of high quality.

### Statistical analysis

Meta-analysis of the data from the included articles was performed using RevMan 5.4 software. Continuous variables were expressed as mean difference (MD) and dichotomous variables as odds ratio (OR), and the size of each pooled effect size and its 95% confidence interval (CI) were calculated. Heterogeneity was analyzed using the Chi-square test, and the size of heterogeneity was judged based on the *I*^*2*^ value. When *P* > 0.1 or *I*^*2*^ ≤ 50%, heterogeneity between studies was not significant and fixed effect model was used for analysis; if *P* ≤ 0.1 or *I*^*2*^ > 50%, heterogeneity between studies was significant, and random effect model was used for analysis. To investigate the potential for publication bias for each risk factor, we employed Egger’s test, which examines the relationship between the effect sizes and their standard errors. A P value of less than 0.1 in this context suggested a statistically significant difference, indicating potential bias.

## Results

### Literature screening procedure and results

**Fig. 1:**
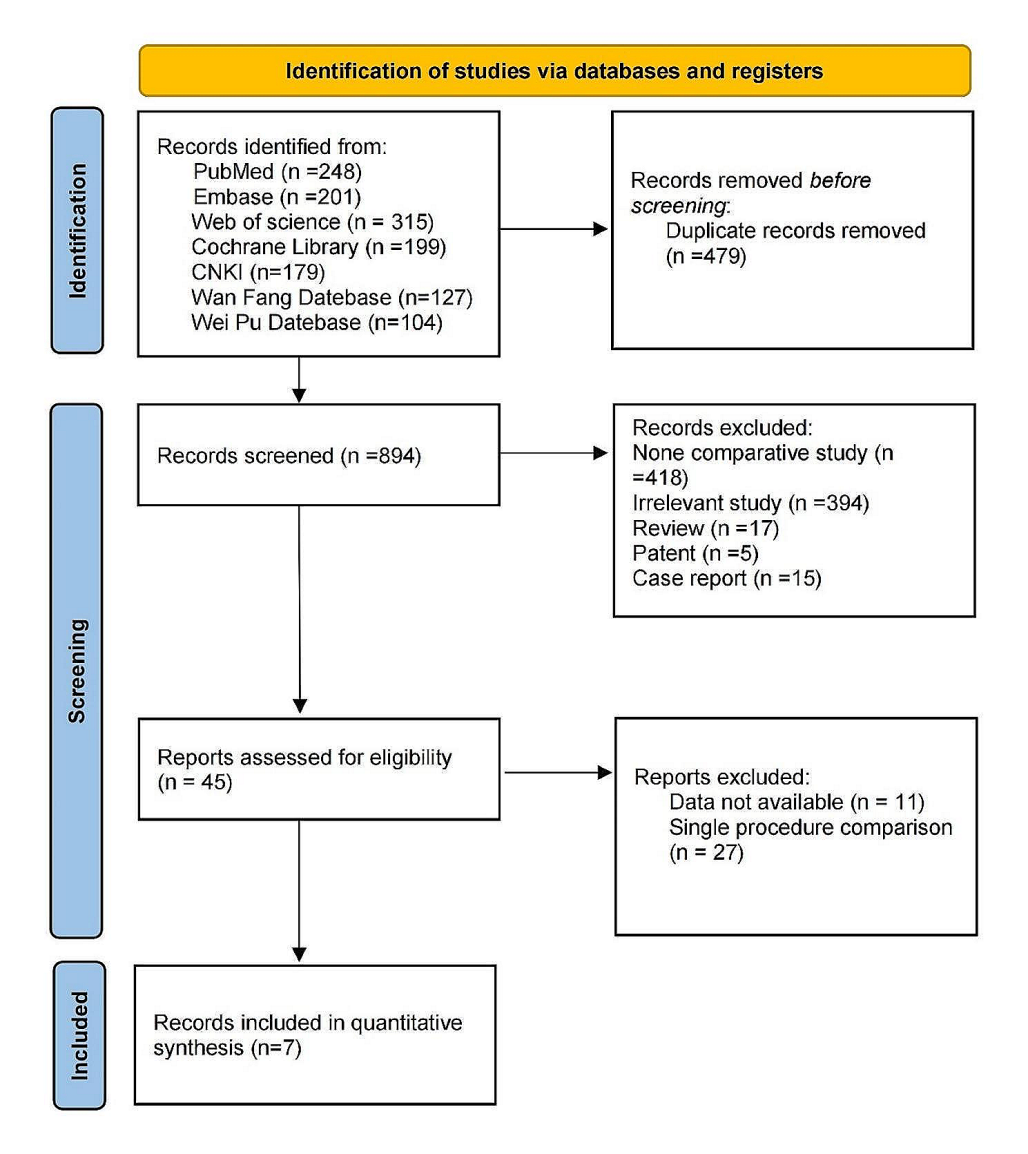
Flowchart illustrating the literature search and the selection of included studies

In this study, 1373 papers were obtained through a preliminary search, 479 repeated publications were eliminated by software, titles, and abstracts were read, and 849 papers that obviously did not meet the inclusion criteria were eliminated. After careful reading of the full text and quality evaluation, 38 unqualified papers were further excluded, and 7 qualified papers [8–14] were finally included. The paper screening process is presented in Fig. 1. A total of 420 patients were included, including 188 patients in the CBT group and 232 patients in the PS group. The main characteristics of the included studies are presented in Table 1. The baseline data of patients in the 7 included literatures showed no statistical difference, with comparability.

**Table 1:** The basic characteristics of the included studies

Study	Study Design	Country	Group	*n*	Age(years)	*N*(Male/Female)	Bone mineral density (SD)	Operation Level(*n*)	Follow up(Month)	Complication(*n*)
Lee,2022	Retrospective	Korea	CBT	22	62.7 ± 10.1	9/13	NR	NR	12	None
			PS	31	64.2 ± 9.3	12/19	NR	NR	12	None
Yang,2023	Retrospective	China	CBT	21	58.51 ± 9.94	11/10	NR	NR	NR	NR
			PS	23	59.28 ± 10.37	13/10	NR		NR	NR
Chen,2021	Retrospective	China	CBT	28	69.35 ± 5.53	14/14	-2.71 ± 0.18	NR	12.68 ± 2.91	Screw perforates cortex(3)
			PS	32	67.28 ± 5.75	18/14	-2.72 ± 0.23	NR		Mild anemia(4); Low back soreness(3)
Ma,2022	Retrospective	China	CBT	20	56.1 ± 12.7	10/14	NR	L2-3(6);L3-4(9); L4-5(7)	NR	Dural tear(1); Incision infection(2);
			PS	24	55.5 ± 11.8	17/15	NR	L2-3(6);L3-4(1); L4-5(9)	NR	Dural tear(3); Incision infection(2);
Guo,2022	Retrospective	China	CBT	53	54.96 ± 5.51	18/35	NR	T12-L1(9);L1-2(10);L2-3(11);L3-4(18);L4-5(5)	16.6 ± 0.48	Delayed wound healing(3)
			PS	61	55.26 ± 5.32	20/41	NR	T12-L1(10);L1-2(14);L2-3(16);L3-4(13);L4-5(8)		Delayed wound healing(4)
Zhong,2022	Randomized clinical trial	China	CBT	30	65.43 ± 9.14	13/17	-1.95 ± 0.73	NR	3	NR
			PS	30	66.06 ± 9.28	14/16	-2.01 ± 0.57	NR	3	NR
Li,2023	Retrospective	China	CBT	9	61.78 ± 5.56	6/3	NR	L3-4(2);L4-5(3);L5-S1(4)	5.18 ± 0.92	Screw dislocation(1)
			PS	20	61.85 ± 5.58	9/11	NR	L3-4(3);L4-5(9);L5-S1(8)	4.98 ± 0.52	Dural tear(1)

CBT = Cortical bone trajectory; PS = Pedicle screw; NR = Not reported

### Quality analysis of included studies

**Fig. 2:**
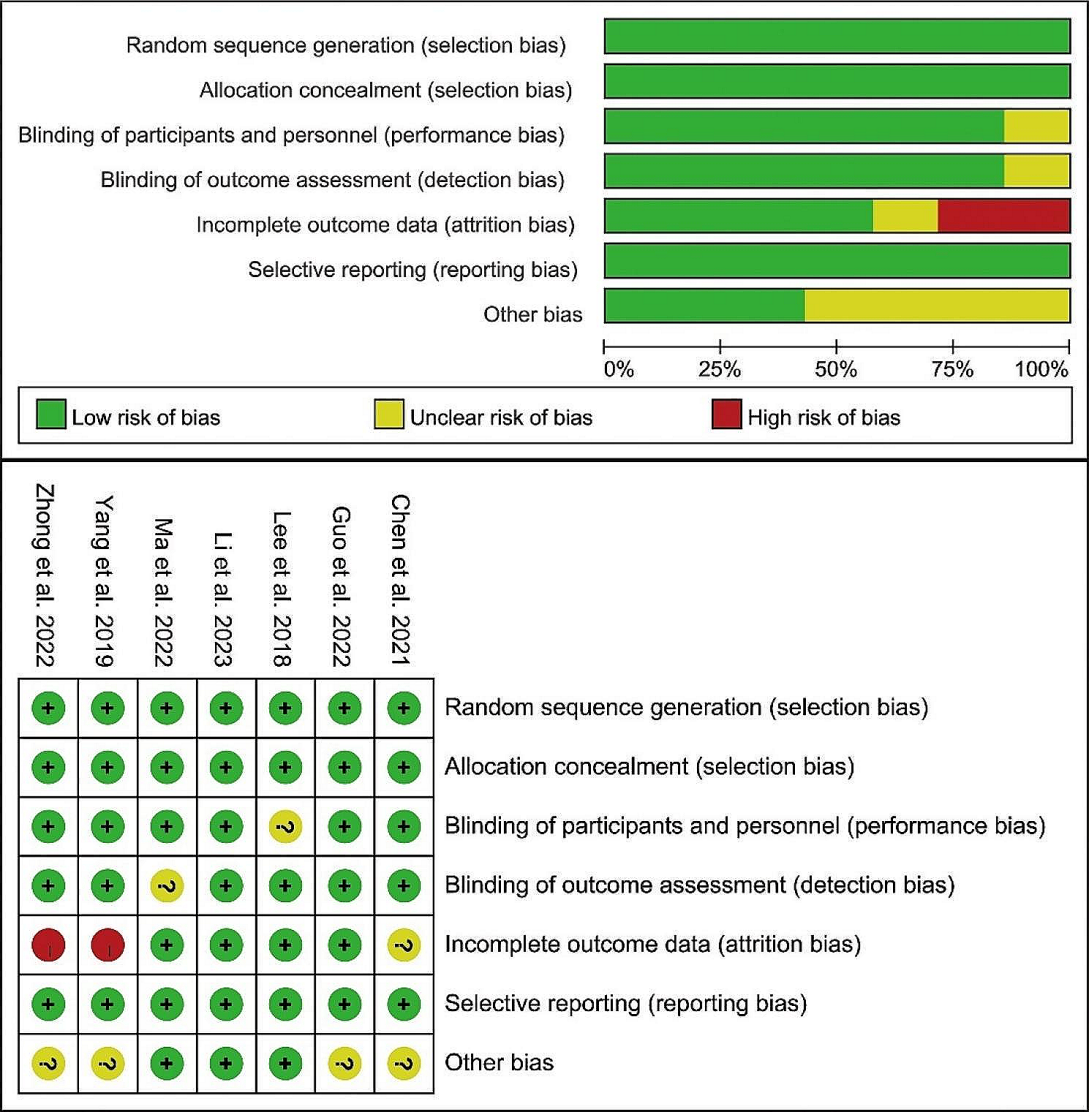
Risk of bias graph for each included study

Risk assessment for the 7 studies included in the analysis was conducted using the Cochrane Risk of Bias tool and is presented in Fig. 2. The quality of non-randomized controlled trials was assessed using the Newcastle-Ottawa Scale (NOS). All included studies scored between 7 and 9 points, indicating high quality. Table 2 provides a summary of the quality scores for each study.

**Table 2:** Quality assessment using the Newcastle-Ottawa Quality Assessment Scale for each none randomized controlled trial

Variable	Lee,2022	Yang,2023	Chen,2021	Ma,2022	Guo,2022	Zhong,2022	Li,2023
Selection							
Representativeness of exposed cohort	1	1	1	1	1	1	1
Selection of nonexposed cohort	1	1	1	1	1	1	1
Ascertainment of exposure	1	1	1		1	1	1
Demonstration that outcome of interest was not present at start of study	1	1	1	1	1	1	1
**Comparability**							
Study controlled for age or gender	1	1	1	1	1	1	1
Study controlled for any additional factor	1			1	1		1
**Outcome**							
Assessment of outcome	1		1	1	1	1	1
Follow-up long enough for outcomes to occur	1	1	1	1	1	1	1
Adequacy of follow-up of cohort	1	1	1	1	1	1	1
Total	9	7	8	8	9	8	9

### Meta-analysis results

#### Operation time

**Fig. 3:**
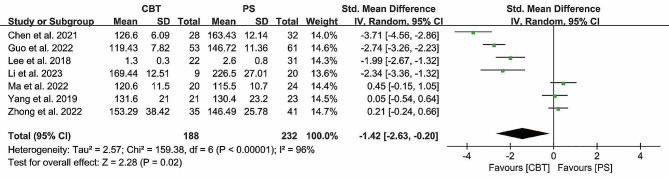
Forest plot of operation time

A total of 7 studies used operation time as an outcome measure, with 188 patients in the CBT group and 232 patients in the PS group. The heterogeneity test (*P* < 0.00001, *I²*= 96%), suggested that there was significant heterogeneity between the studies, and a meta-analysis using a random-effects model showed that: [MD = -1.42, 95% CI (-2.63, -0.20), *P* = 0.02] (Fig. 3), The results showed that the operation time was longer in PS compared to CBT.

#### Intraoperative blood loss

**Fig. 4:**
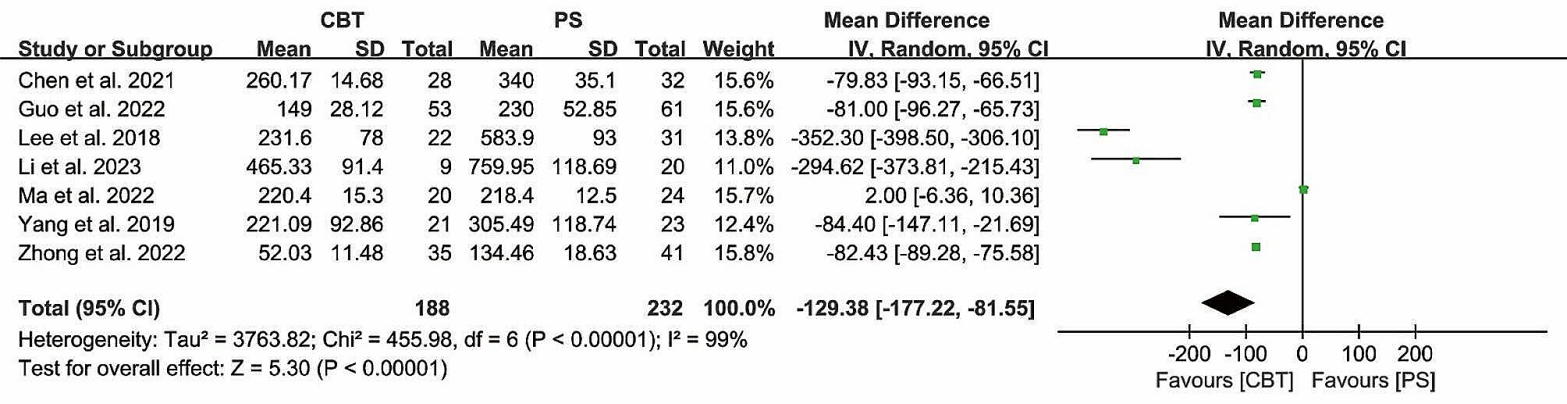
Forest plot of intraoperative blood loss

Intraoperative blood loss was counted in 7 studies, with 188 patients in the CBT group and 232 patients in the PS group. The heterogeneity test (*P* < 0.00001, *I*²= 99%), suggested that there was significant heterogeneity between the studies. The results showed that intraoperative blood loss in the CBT group was significantly lower than that in the PS group [MD = -129.38, 95% CI (-177.22, -81.55), *P* < 0.00001] (Fig. 4), indicating that CBT had a certain effect on the reduction of intraoperative blood loss in patients.

#### Pain evaluation

Preoperative back VAS scores were reported in 6 papers, and heterogeneity test results showed *P* = 0.33; *I²*= 13%. The results showed that there was no significant difference in preoperative back VAS score between CBT and PS group [MD = 0.01, 95% CI (-0.20, 0.23), *P* = 0.90]. There was no heterogeneity in the study. Preoperative leg VAS scores were reported in 6 papers, and heterogeneity test results showed *P* = 0.53; *I²*= 0%. The results showed that there was no significant difference in preoperative leg VAS score between CBT and PS group [MD = -0.04, 95% CI (-0.24, 0.17), *P* = 0.72]. Both groups were comparable.

**Fig. 5:**
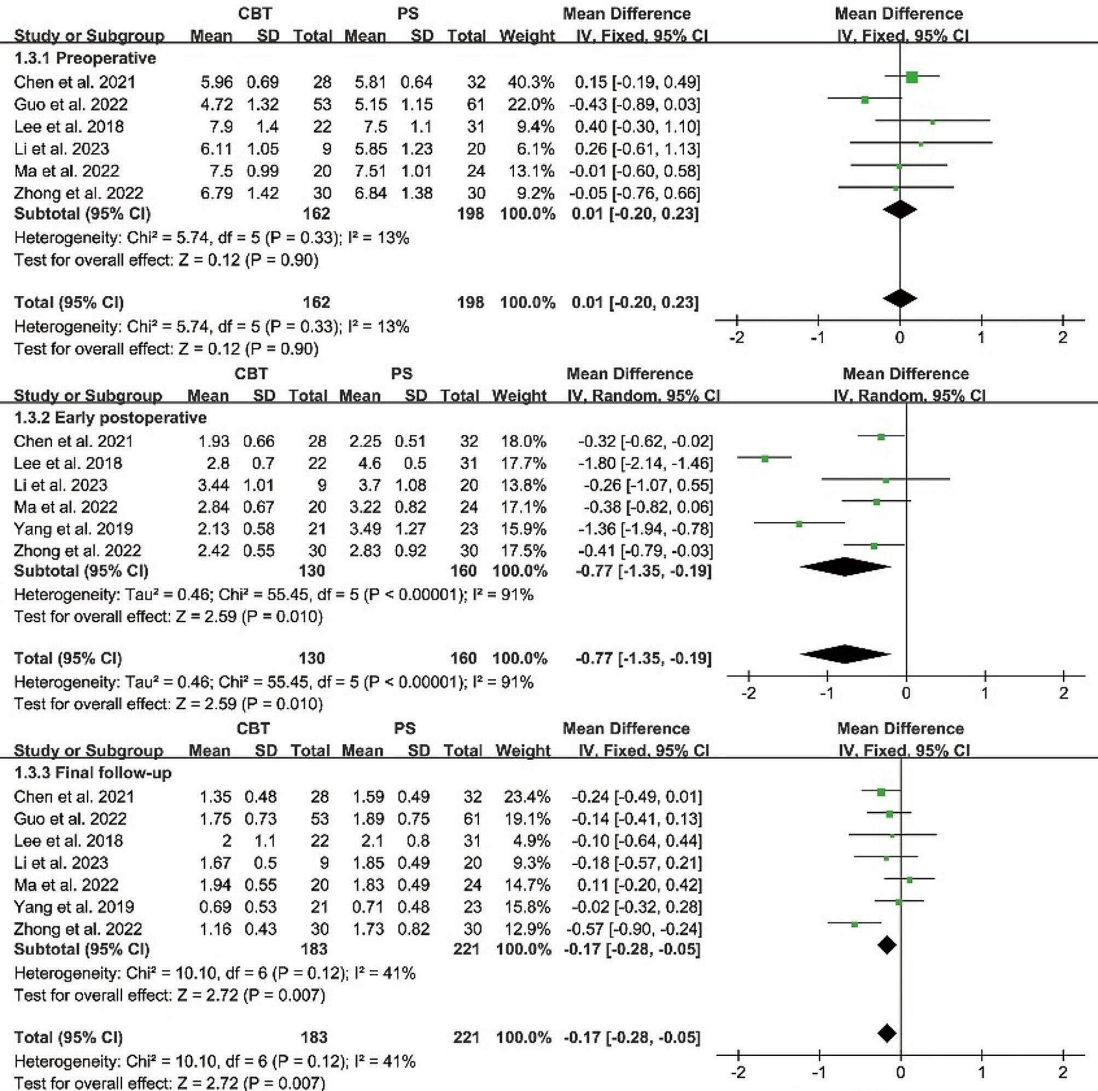
Forest plot of back VAS

**Fig. 6:**
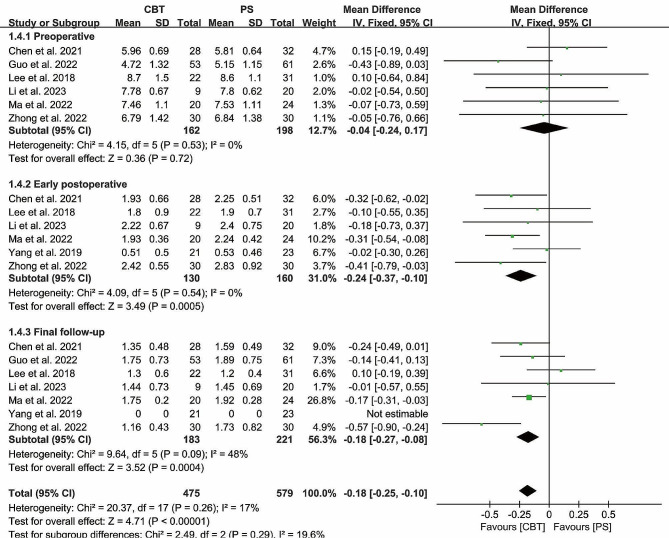
Forest plot of leg VAS

Back VAS scores at early postoperative were reported in 6 papers, and heterogeneity test results showed *P* < 0.00001; *I²*= 91%. The results showed that back VAS score at early postoperative in CBT group was significantly lower than that in PS group [MD = -0.77, 95% CI (-1.35, -0.19), *P* = 0.01]. Leg VAS scores at early postoperative were reported in 6 papers, and heterogeneity test results showed *P* = 0.54; *I²*= 0%. The results showed that leg VAS score at early postoperative in CBT group was significantly lower than that in PS group [MD = -0.24, 95% CI (-0.37, -0.10), *P* = 0.0005] (Figs. 5 and 6). At the same time, lower back and leg pain relief at the last follow-up was also better in the CBT group than in the PS group.

#### Oswestry disability index

Preoperative Oswestry disability index were reported in 6 papers, and heterogeneity test results showed *P* = 0.79; *I²*= 0%. The results showed that there was no significant difference in preoperative Oswestry disability index between CBT and PS group [MD = 0.41, 95% CI (-0.46, 1.28), *P* = 0.36]. There was no heterogeneity in the study.

**Fig. 7:**
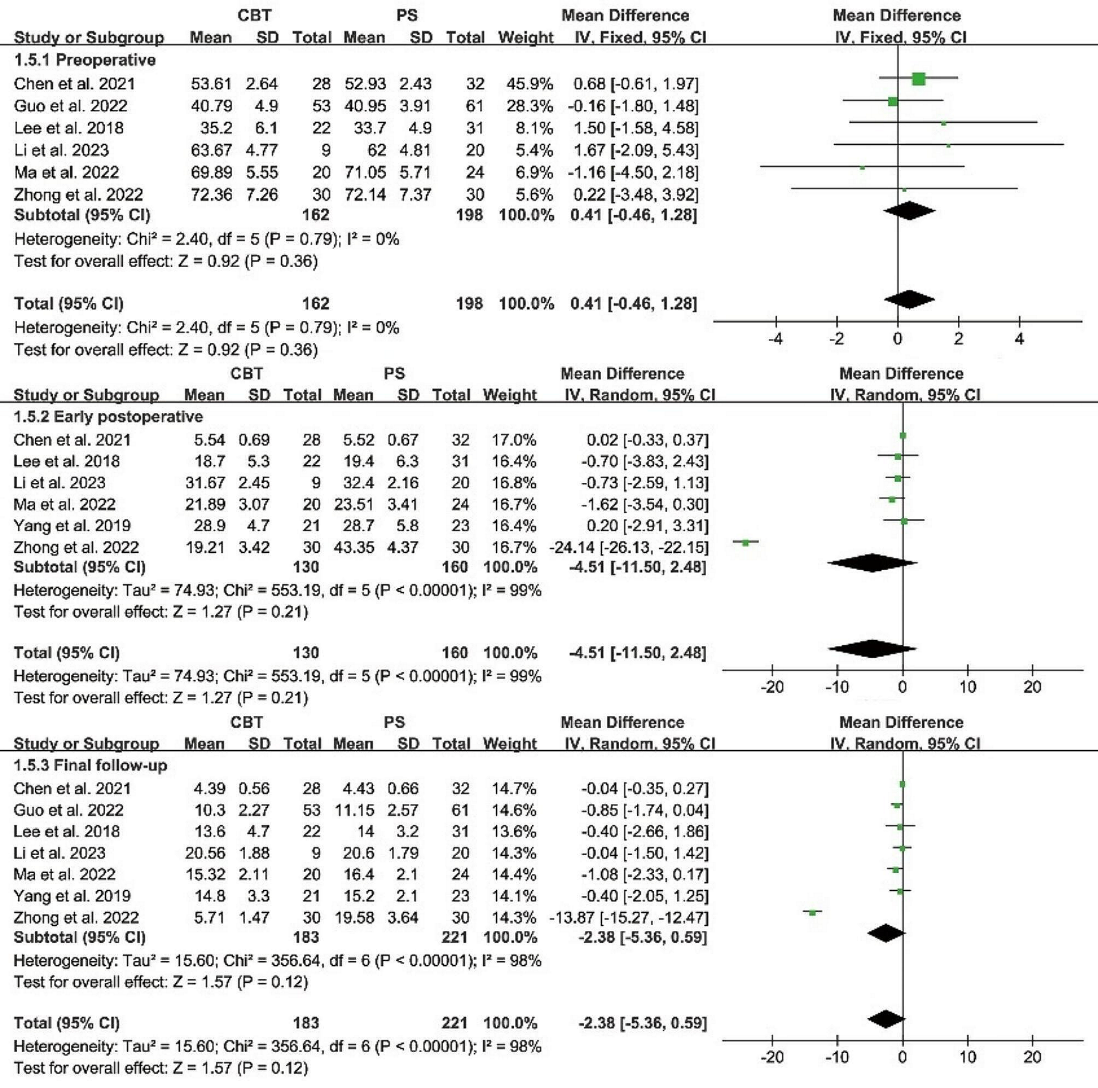
Forest plot of ODI

Postoperative Oswestry disability index was reported in 6 papers, and heterogeneity test results showed *P* < 0.00001; *I²*= 99%. The results showed that there was no significant difference in the preoperative Oswestry disability index between CBT and PS group [MD = -4.51, 95% CI (-11.50, 2.48), *P* = 0.21]. (Fig. 7). Similar to the early postoperative period, the ODI index remained statistically insignificant between the two groups at final follow-up [MD = -2.38, 95% CI (-5.36, 0.59), *P* = 0.12].

#### Disc height

Preoperative disc height were reported in 3 papers, and heterogeneity test results showed *P* = 0.51; *I²*= 0%. The results showed that there was no significant difference in preoperative disc height between CBT and PS group [MD = 0.01, 95% CI (-0.33, 0.34), *P* = 0.98]. Both groups were comparable.

Preoperative disc height was reported in 3 papers, and heterogeneity test results showed *P* = 0.51; *I²*= 0%. The results showed that there was no significant difference in preoperative disc height between CBT and PS group [MD = 0.01, 95% CI (-0.33, 0.34), *P* = 0.98].

**Fig. 8:**
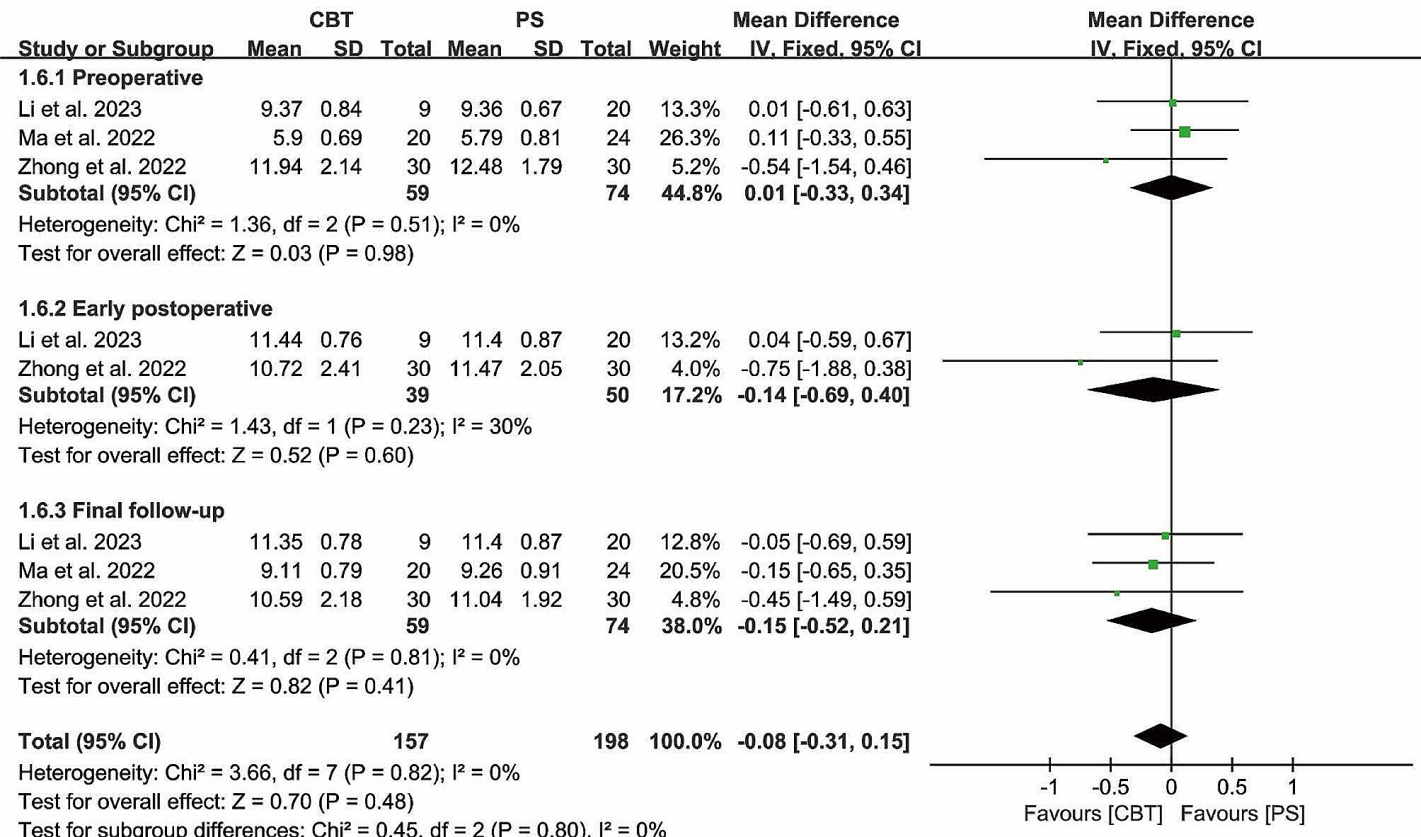
Forest plot of DH

Final follow-up disc height was reported in 3 papers, and heterogeneity test results showed *P* = 0.81; *I²*= 0%. The results showed that there was no significant difference in postoperative disc height between CBT and PS group [MD = -0.15, 95% CI (-0.52 0.21), *P* = 0.41] (Fig. 8).

#### Hospital length stay

**Fig. 9:**
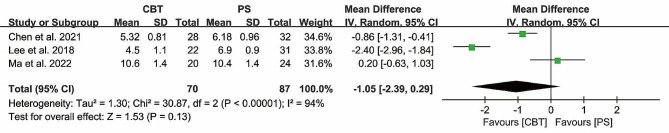
Forest plot of hospital length stay

Hospital length stay was reported in 3 papers, heterogeneity test result *P* < 0.00001; *I²*=94%. There was significant heterogeneity across the studies. The results showed that there was no significant difference in hospital length stay between CBT and PS group [MD = -1.05, 95% CI (-2.39, 0.29), *P* = 0.13] (Fig. 9).

#### Complications

**Fig. 10:**
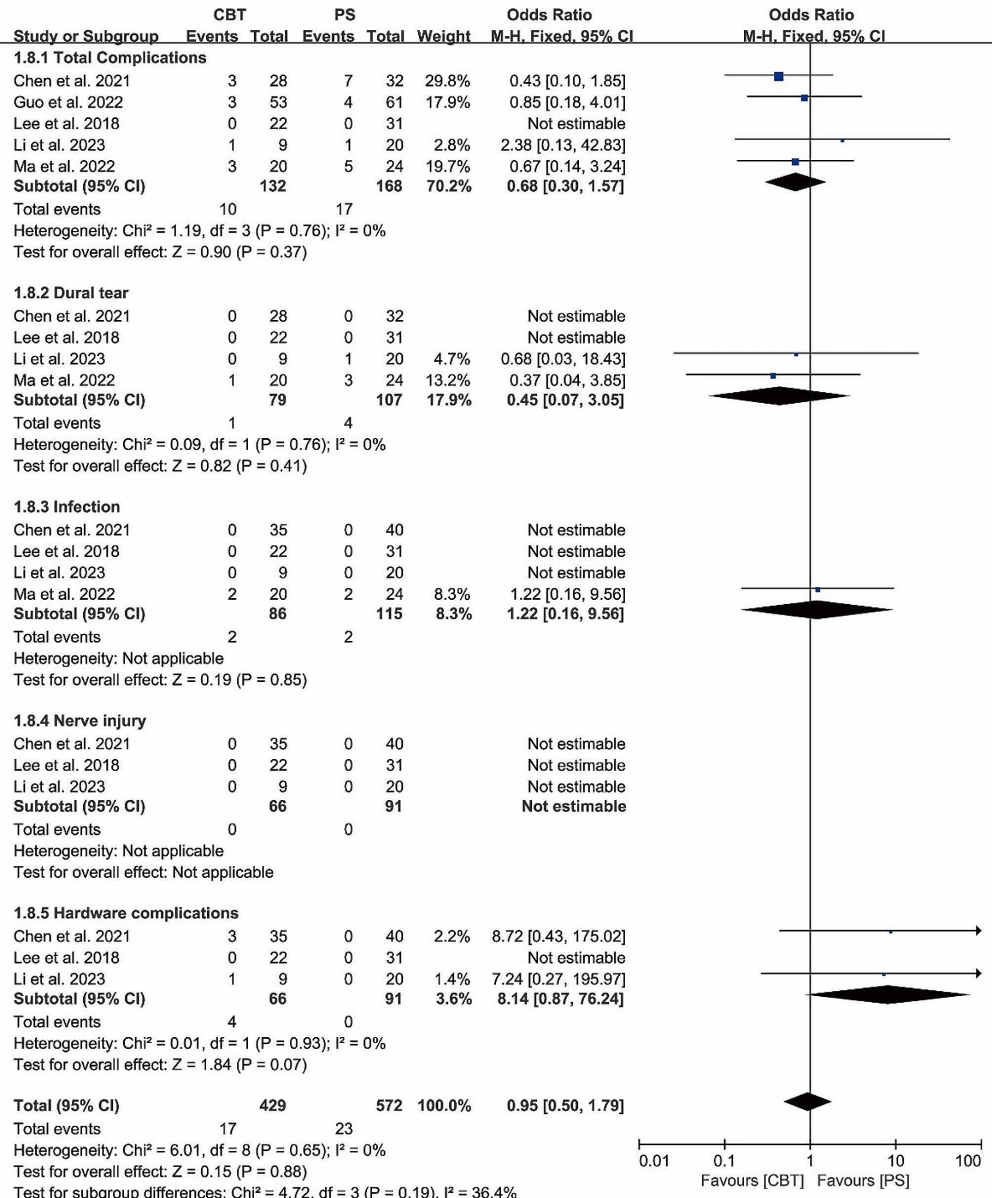
Forest plot of the number of complications

Total complications were reported in 5 papers, and heterogeneity test results showed *P* = 0.76; *I²*= 0%. The results showed that there was no significant difference in total complications between CBT and PS group [OR = 0.68, 95% CI (0.30, 1.57), *P* = 0.37]. Dural tears were reported in 4 papers, and heterogeneity test results showed *P* = 0.76; *I²*= 0%. The results showed that there was no significant difference in dural tears between CBT and PS group [OR = 0.45, 95% CI (0.07, 3.05), *P* = 0.41]. Hardware complications were reported in 3 papers, and heterogeneity test results showed *P* = 0.93; *I²*= 0%. The results showed that there was no significant difference in hardware complications between CBT and PS group [OR = 8.41, 95% CI (0.87, 76.24), *P* = 0.07]. (Fig. 10).

#### Heterogeneity and sensitivity analysis

The analysis indicated significant variability in intraoperative blood loss and operation duration. To evaluate how this variability affected the results, a sensitivity analysis was performed by excluding individual studies from the operation time assessment. The outcomes of this analysis aligned with the original findings, implying that the heterogeneity had a negligible impact on the study conclusions. Potential contributors to this variability include surgeons vary in experience and operating habits, proficiency in surgical technique, recording method of operation time and intraoperative blood loss, accuracy and completeness of medical record recording. The surgeon ‘s learning curve regarding CBT screw use may have influenced the results to some extent.

#### Publication deviation

**Fig. 11:**
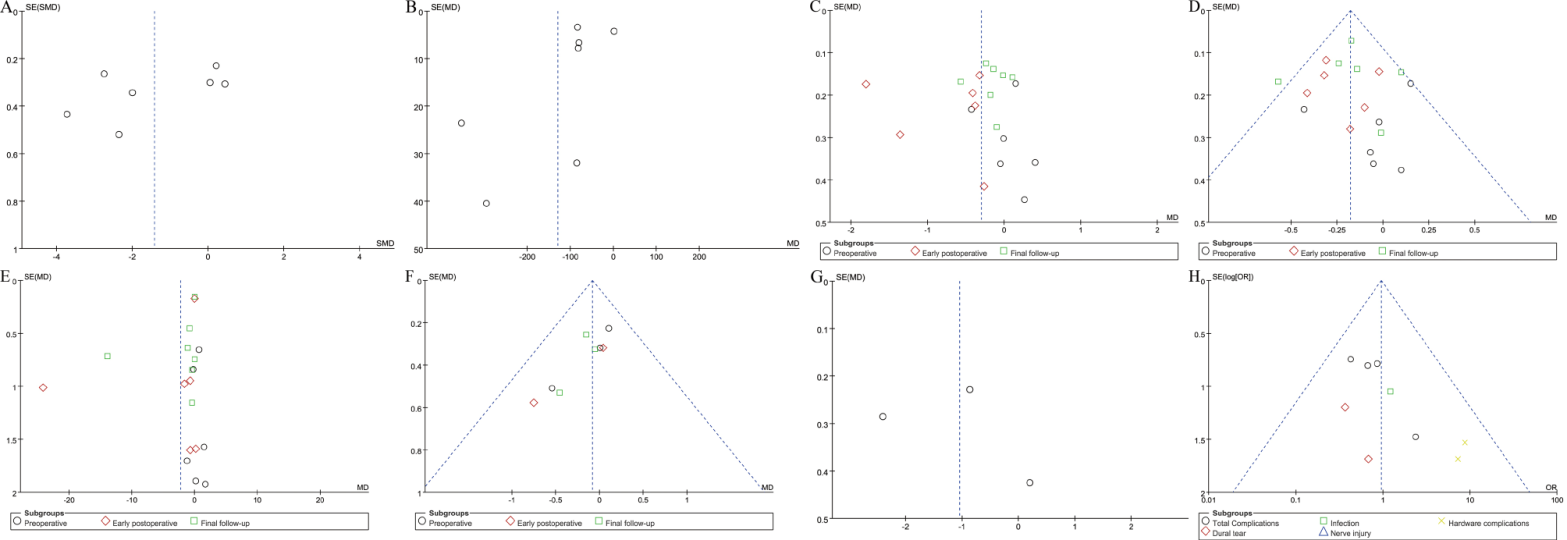
Funnel plot of publication bias for operation time(**A**), intraoperative blood loss(**B**), back VAS(**C**), leg VAS(**D**), ODI(**E**), DH(**F**), hospital length stay(**G**), complications(**H**)

The study included 7 articles and tested all outcome measures for publication bias. The funnel plot was visually assessed for each outcome measure, and it appeared to be mostly symmetrical, indicating a low likelihood of publication bias. Figure 11 provide supporting evidence for this finding. In addition, we performed tests for bias regarding operation time, intraoperative blood loss. The results of Egger’s test are shown in supplementary Figs. 12–13.

**Fig. 12:**
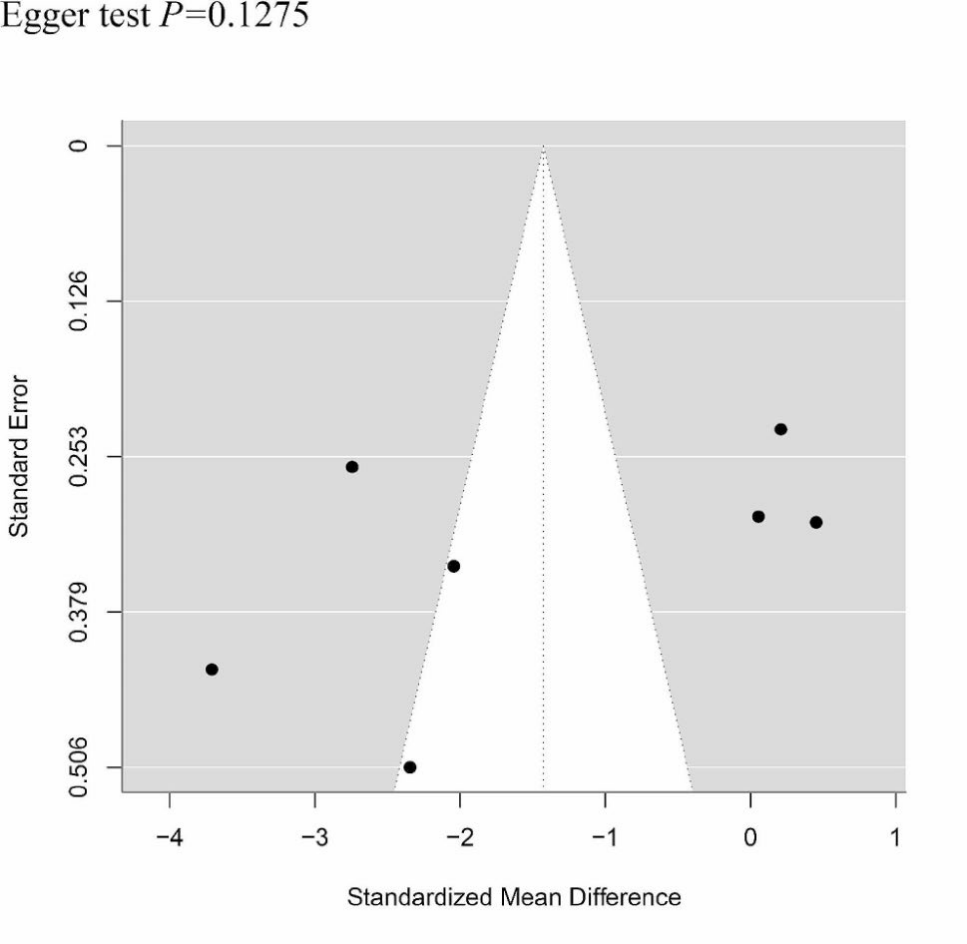
Funnel plot of Egger test for operation time

**Fig. 13:**
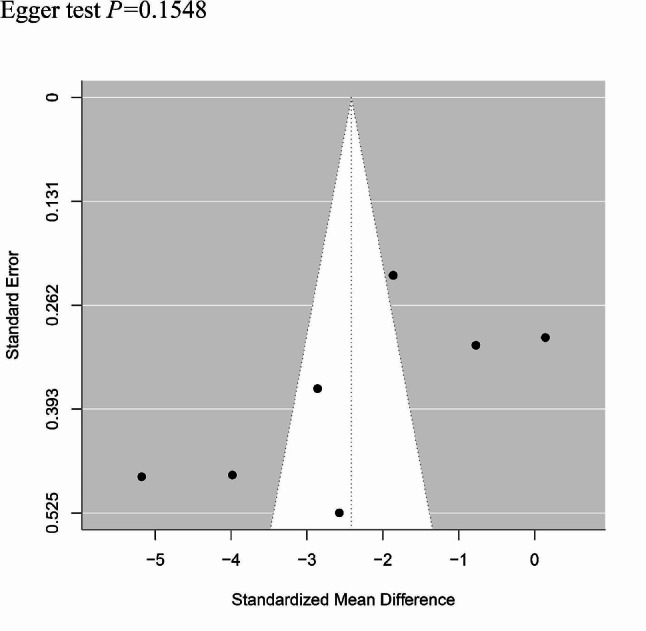
Funnel plot of Egger test for intraoperative blood loss

## Discussion

ASD after lumbar fusion is a great concern and accounts for a significant proportion of spinal revision surgeries. The risk of asymptomatic degenerative changes following lumbar fusion has been reported to be as high as 57%, while the probability of developing symptomatic ASD ranges from 1.9–30.3% [15–17]. The pathological process of ASD mainly includes lumbar disc herniation at adjacent segments, spinal stenosis, instability of adjacent segments, lumbar scoliosis at adjacent segments, and vertebral compression fractures after lumbar fusion, and its main pathogenesis is that internal fixation fusion limits the normal activity of the spine while changing the biomechanics of adjacent segments at the fusion level and accelerating the degeneration process of adjacent segments [3]. Conservative treatment is mostly selected in the early stage of adjacent segment diseases after lumbar fusion; if conservative treatment is ineffective, surgical treatment is recommended for patients with intractable low back pain or progressive nerve damage [18]. The indications for surgery for adjacent segment disease are in principle the same as for other lumbar lesions. How select reasonable surgical methods such as simple nucleus pulposus removal, spinal canal decompression or fixed fusion should not only refer to the type of adjacent segment disease, the severity of degeneration, and the stability of the diseased segment, but also combine with individual factors such as the patient’s age, body mass index, bone conditions, and economic ability. At the same time, the specific treatment methods and internal fixation options should also be considered according to the previous surgical conditions and whether there is internal fixation retention [19].

The traditional pedicle screw technique allows the screw to penetrate through the three columns of the vertebral body and achieve three-column fixation of the vertebral body, which is characterized by stability, good orthopedic effect, and high fusion rate [20–22]. This technique has been widely used in the treatment of spinal degenerative diseases, tumors, trauma, and infections in recent years. However, for osteoporotic patients, screw loosening and internal fixation failure occur at a high rate [23]. For patients with developed or obese low back muscles, extensive dissection of the back musculature is often required for convenient screw placement, which easily causes postoperative low back pain and affects the prognosis [24]. To address these issues, Santoni et al. [5]. presented the cortical bone trajectory (CBT) screw technique in which the trajectory contacted four cortices, i.e., the cortex at the dorsal entry point, the medial cortex of the posterior pedicle wall, the lateral cortex of the anterior pedicle wall, and the vertebral cortex, maximizing contact with the cortical bone, improving screw holding strength, and then optimizing the biomechanical properties of the screw; the entry point closer to the midline reduced dissection of the back musculature, reduced surgical trauma, and improved prognosis. Fixation with CBT screws increases the contact area between the screw and cortical bone by having the threaded pitch more proximally than caudally [25–27]. In addition, tissue dissection is often difficult in obese or low back muscle-developed patients undergoing traditional posterior lumbar fusion. The CBT screw technique had an entry point closer to the midline avoiding extensive muscle dissection, protected nerves innervating the facet joints and multifidus muscle, and reduced postoperative low back pain due to lipidation of the paravertebral muscle [28]. Multiple studies have demonstrated that this technique is a valid alternative to traditional pedicle screw techniques [26, 29–31].

The rate of screw loosening may increase if the original trajectory is reinserted using the same instrumentation as in the initial surgery [32, 33]. The course of the disease from the initial lumbar fusion to ASD is generally very long, and the bone quality of such patients is decreased to varying degrees. In their posterior lumbar decompression, fusion, and internal fixation surgery, if the traditional PS is used, the screw loosening rate will inevitably increase, and loosening of the screw-bone interface may occur in the long term, which leads to screw pullout, internal fixation failure and junctional kyphosis [11]. In addition, patients with adjacent vertebral disease tend to have a very high average age at readmission for low back and leg pain, and according to the initial surgical plan, the internal fixation device for the initial surgery needs to be removed, so that the incision length of the operation is longer and the blood loss will be more than that of the initial operation, and factors such as increased age and increased surgical incision inevitably increase the patient’s surgical risk.

The unique trajectory also makes it possible to perform CBT screw and conventional pedicle screw placement in the same pedicle. Mullin et al. [34]. demonstrated the feasibility of dual trajectory fixation with simultaneous placement of conventional pedicle screws and CBT screws at all levels of the lumbar spine by performing CT scans and reconstructions of the lumbar spine in 47 patients. CBT internal fixation technique and PS internal fixation technique screw trajectory is different, no need to operate the original surgical segment, the same can decompress the adjacent segment, bone graft, fusion, internal fixation operation, and can achieve the same surgical results. Therefore, when secondary revision surgery is performed for lumbar adjacent spondylosis (ASD), the original screws may not be removed, which reduces the operation time and blood loss, and the difficulty of surgery is also greatly reduced [32, 35, 36]. Chen et al. [32]. treated 6 cases of lumbar ASD with CBT screw technique without removing preexisting devices and combined with minimally invasive fusion surgery to reduce wound length, blood loss, and soft tissue damage. Rodriguez et al. [37]. revised 5 patients with adjacent spondylosis who had a previous history of lumbar fusion and placed CBT screws using CT navigation without removing the original internal fixation, combined with posterior decompression, to reduce the operation time, blood loss, and exposure range, with an average hospital stay of 2.8 days, no surgical complications, and good improvement of patients’ symptoms, and Kotheeranurak et al. [38]. selected unilateral CBT screw fixation plus endoscopy-assisted anterior fusion for patients with degeneration of L5-S1 after L4-L5 spondylolisthesis, which was successfully placed under navigation despite the presence of pedicle screws at L5. Compared with traditional PS internal fixation, CBT internal fixation has the advantages of less paravertebral muscle dissection, less superior facet and mastoid invasion, and strong screw holding power; in addition, compared with traditional PS fixation, CBT screws have smaller diameter, smaller incision during screw implantation, less muscle tissue dissection, less intraoperative blood loss, lower postoperative infection rate, and faster patient recovery, reflecting the concept of minimally invasive and rapid rehabilitation [11].

At present, the indications for the application of CBT internal fixation techniques include: (1) patients with lumbar degenerative diseases, such as combined osteoporosis fixation effect is better; (2) patients with obesity, low back muscle development and high iliac spine; (3) adjacent vertebral diseases after pedicle screw trajectory screw placement; (4) remedial screw placement after pedicle screw loosening and pullout and fixation failure; (5) diseases mainly caused by anterior and middle vertebral column destruction, such as lumbar tuberculosis and intervertebral space infection. This technique also has the following disadvantages: (1) lack of CBT screw anchoring point or screw path has been damaged such as lumbar spondylolisthesis, previous surgery or bone destructive disease resulting in bone destruction or absence in the lamina or isthmus region; (2) high technical requirements for screw implantation, difficult re-diversion when the screw path direction is wrong, and the risk of isthmus and pedicle fracture cannot be avoided; (3) there is a risk of upper and lower nerve root injury at the same time; (4) difficult screw and rod installation during long-segment fixation; (5) long learning curve, low accuracy of freehand screw placement, and easy screw puncture of the pedicle and vertebral cortex during screw placement. With the advent of new auxiliary techniques such as spinal robotics, navigation, 3D guide navigation, and three-dimensional CT preoperative trajectory planning, more options have been provided for auxiliary screw implantation [11, 39]. Studies have shown that CBT screw placement assisted by spine robotics, navigation, 3D guide navigation is more accurate and safer than free-hand screw placement [28, 40]. Therefore, neoadjuvant screw placement technique can solve the problems of difficult screw placement and low accuracy of CBT screws, so CBT, as a minimally invasive screw placement method, is worth recommending in the treatment of adjacent vertebral diseases.

For the selection of posterior internal fixation and the treatment methods of original internal fixation, it is recommended to use the following: (1) preserve the original internal fixation, use cortical screw fixation for the new fusion level, which does not interfere with each other, and have less trauma and good stability for cortical screw fixation; (2) use the original internal fixation for the original internal fixation that has loosened or has signs of loosening, affect the cortical screw placement installation, those who need to use the original adjacent group of screws, and those who have requirements of the patient or their family members; (3) use of cortical screws is recommended for those who have severe osteoporosis and have significantly enlarged original pedicle orifice and are not suitable for the use of pedicle screws and original pedicle screw retention [19].

Limitations of this study are that most of the included articles were retrospective studies, only one was prospective for reference, and long-term follow-up was lacking to fully evaluate the safety and efficacy of this technique. As one of the very important evaluation indexes of spinal fusion, only two of the seven articles included in this manuscript have made relevant reports and cannot be compared, and the evaluation of this index should be focused on in future studies. In addition, CBT instrumentation is a novel screw placement technique, and the learning curve may influence the results of the study. Therefore, subsequent prospective studies with large samples and multiple centers are needed to obtain higher levels of evidence support.

## Conclusion

In summary, compared with the traditional PS internal fixation technique, CBT screw internal fixation technique can achieve the same clinical effect in the treatment of ASD, and has the advantages of less exposure range of the surgical area, less blood loss, shorter operation time, less early postoperative pain, and strong screw purchase force.

### Electronic supplementary material

Below is the link to the electronic supplementary material.

Supplementary Material 1

## Data Availability

The data sets generated and analyzed during the current study are not publicly available but can be obtained from the corresponding author on reasonable request.
